# Composition and Rheological Properties of Polysaccharide Extracted from Tamarind (*Tamarindus indica* L.) Seed

**DOI:** 10.3390/molecules24071218

**Published:** 2019-03-28

**Authors:** Huimin Shao, Hui Zhang, Yanjun Tian, Zibo Song, Phoency F. H. Lai, Lianzhong Ai

**Affiliations:** 1 Shanghai Engineering Research Center of Food Microbiology, School of Medical Instruments and Food Engineering, University of Shanghai for Science and Technology, Shanghai 200093, China; ncuskshaohuimin@163.com (H.S.); zhh8672@126.com (H.Z.); plai856@hotmail.com (P.F.H.L.); 2 Shandong Food Ferment Industry Research & Design Institute, Jinan 250013, China; tianyanjun16@163.com; 3 Yunnan Maodouli Group Food Co., Ltd., Yuxi 653100, China; Szb1031@163.com

**Keywords:** tamarind seed polysaccharide, chemical composition, rheological properties, pH-resistance, thermo-stability

## Abstract

A polysaccharide was extracted in high yield from tamarind (*Tamarindus indica* L.) seed (TSP) by acidic hot water extraction and ethanol precipitation. It was composed of 86.2% neutral polysaccharide, 5.4% uronic acid and 1.3% protein. The molecular weight of TSP was estimated to be about 1735 kDa, with glucose, xylose, and galactose in a molar ratio of 2.9:1.8:1.0 as the major monosaccharides. The steady shear and viscoelastic properties of TSP aqueous solutions were investigated by dynamic rheometry. Results revealed that TSP aqueous solution at a concentration above 0.5% (*w*/*v*) exhibited non-Newtonian shear-thinning behavior. Dynamic oscillatory analysis revealed that 10% (*w*/*v*) TSP showed as a “weak gel” structure. Apparent viscosities and viscoelastic parameters of TSP solutions decreased drastically in an alkaline solution of pH > 10, but slightly influenced by acidic solution, high temperature and the presence of salt ions and sucrose. These results indicated that TSP possessed excellent pH-resistance and thermo-stability, which might be suitable for applications in acidic beverages and high-temperature processed foodstuffs.

## 1. Introduction

Tamarind (*Tamarindus indica* L.) is an evergreen large tree of the genus Tamarindu of the familiy Caesalpinioideae, native to Southeast Asia, India and Equatorial Africa, which are subtropical and tropical regions [1]. Tamarind seed polysaccharide (TSP), known as tamarind gum, is a neutral xyloglucan (XG) extracted from tamarind seed kernels. It is composed of a β-(1,4)-d-glucan backbone with α-(1,6)-d-xylose branches that are partially substituted with β-(1,2)-d-galactose [2,3]. Several studies have investigated the anti-cancer activity of XG as an antitumor factor [4,5], indicating that TSP is potentially a source of bioactive polysaccharide. In addition, the mucoadhesive properties of TSP were also studied for application as a mucoadhesive polymer [6].

TSP can form viscous solutions when dissolved in water, like many plant polysaccharide gums. It has been used as a food thickener because of its excellent physical properties, such as condensation, thickening, emulsification, viscosity, and gelling abilities [7,8]. Being an ideal natural food additive, it can be used in jams, soft candies, jellies and ice-creams to improve the viscosity or gelling properties of products [9].

Some investigations about the rheological properties of XG have been reported. An aqueous solution of XG showed shear-thinning behavior, a characteristic of non-Newtonian fluids, and influenced by a number of parameters including molecular weight and substituents along β-(1,4)-d-glucan backbone [10]. XG shows a gel-like behavior indicated by non-destructive oscillatory measurements, suggesting potential use as a gelling additive and vehicle in medical applications [11]. When part of the galactose substitutents is removed from the XG, it can form a hydrogel that is thermally reversible in dilute aqueous solutions [12]. Addition of XG into gellan gum can reduce the effective concentration of gellan needed for gelation, i.e., a gel network is formed at a gellan concentration much lower than the gelation threshold of gellan alone [13]. Mixtures of starch and XG yield high viscosities and the degree of pseudoplasticity increased with the XG content [14].

The current study aimed to systematically investigate the rheological change and stability of TSP solutions with respect to food processing factors. The factors studied were set over a wide range of levels, for example, TSP concentrations (0.5–10%), pH (1–13), temperature (5–85 °C), salt ions (Na^+^, K^+^, Ca^2+^) and sucrose (10–30%). The chemical components of the studied TSP were also characterized because it was isolated from tamarind seeds collected from Yunnan, China and has not yet been reported before.

## 2. Results and Discussion

### 2.1. Chemical Composition and Molecular Weight Analysis of TSP

The extraction yield of TSP was determined to be 54.6% (*w*/*w*), higher than that reported by Nayak et al. [15]. The yield difference might be due to the extraction method, especially the extraction temperature, and the breed or physiological stage of the tamarind seeds used. Approximately 86.2% of neutral sugar, 5.4% of uronic acid, 1.3% of protein, 6.1% of moisture and 0.3% of ashes were observed in the TSP sample studied, suggesting that the TSP was mainly a neutral polysaccharide with a few impurities. These results are similar to those reported previously [16].

**Figure 1:**
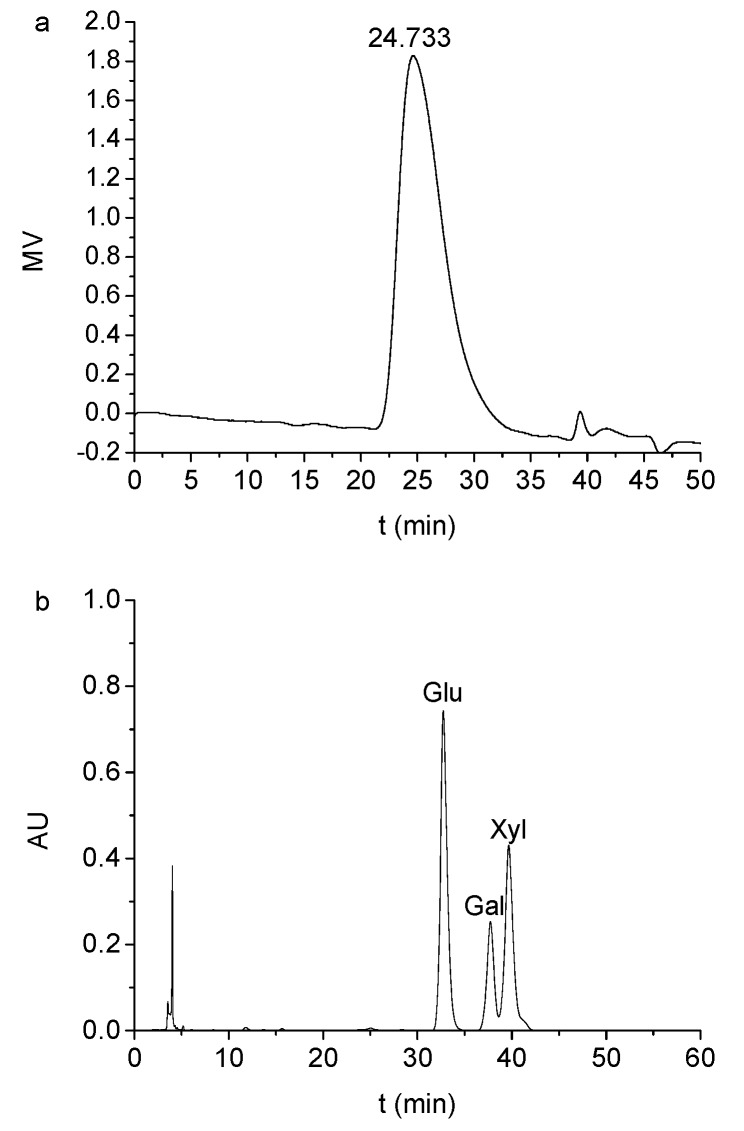
(**a**) The elution curve of TSP solution at 2 mg/mL by HPSEC; (**b**) The elution curve of PMP derivatives of TSP hydrolysate by HPLC.

The mean Mw and distribution of TSP were determined by HPSEC. As shown in Figure 1a, the molecular weight of XG was widely distributed and monodisperse, giving a mean estimated TSP Mw of about 1735 kDa. This result is different from the XGs with multiple fractions of 25, 80, 150, 590 and 1200 kDa obtained by Lima et al. [17]. The difference may be related to the different extraction methods used or the diversity of biological origin. Likely, the molecular weights of tamarind xyloglucan polymers could be associated with the self-association capacity of cells and have an effect on the structural function of primary cell walls. Monosaccharide composition analyses of standard (Figure S1) and sample (Figure 1b) revealed that TSP was a heteropolysaccharide composed of glucose, xylose and galactose in a molar ratio of 2.9:1.8:1.0. This ratio was similar to a previous report that showed a molar ratio of 3:2:1 [18].

### 2.2. FT-IR Spectroscopy

**Figure 2:**
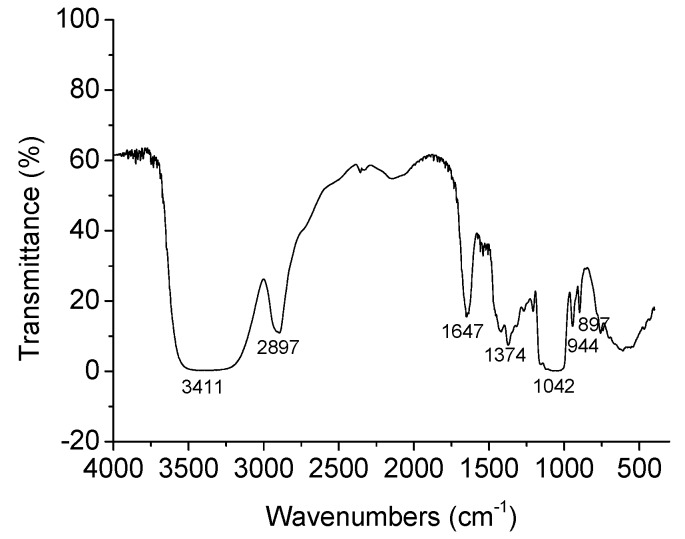
FT-IR spectrum of TSP.

Figure 2 shows the FT-IR spectrum of TSP, which presented peaks typical of the glycosidic structure of xyloglucans. The strong absorption peak at 3411 cm^−1^ represents the hydroxyl (O-H) stretching vibration of polysaccharides and water involved in hydrogen bonding [19]. The absorption peak at 2897 cm^−1^ corresponds to the methylene group (C-H) stretching vibration peak characteristic of polysaccharides [20]. The absorption peaks at 1647 cm^−1^ and 1374 cm^−1^ refer to the presence of carbonyl or carboxylic acid groups (C=O) [21,22]. The absorption peak at 1042 cm^-1^ implies that TSP is pyran-glycosylated [23]. In the anomeric region (950–700 cm^−1^), the polysaccharide exhibited an obvious characteristic absorption at 897 cm^−1^, which was assigned to d-galactopyranose [24]. The absorption peak at 944 cm^−1^ could represent d-glucopyranose.

### 2.3. Flow Behaviors of TSP

**Figure 3:**
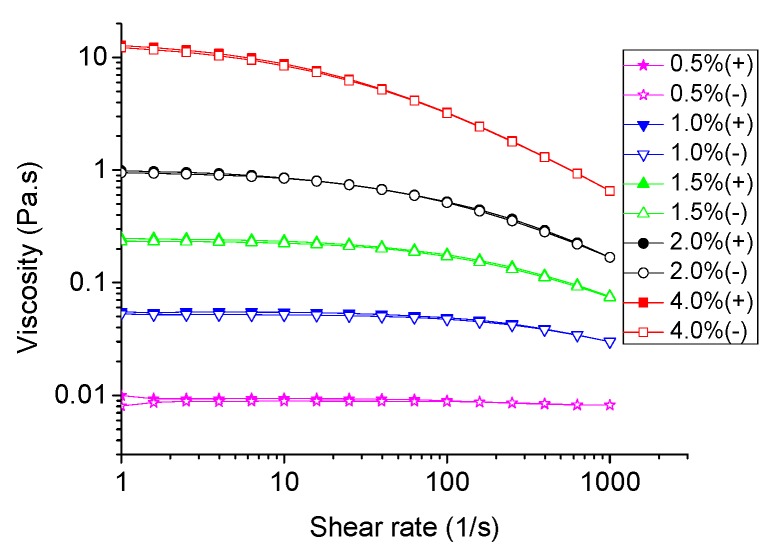
Apparent viscosity changes of TSP at different concentrations (*w*/*v*) at 25 °C with shear rates ranging from 1 to 1000 s^−1^ (solid symbol) and from 1000 to 1 s^−1^ (hollow symbol).

The apparent viscosity of TSP solutions at different concentrations is shown in Figure 3. The results indicated that the apparent viscosity of TSP solutions increased with the increase of concentration, which was consistent with a previous report [25]. The apparent viscosity of TSP solutions showed a Newtonian plateau at low shear rates, and became shear thinning at high shear rates and more concentrated concentrations. At low shear rates, the stretching macromolecules of TSP would intertwine to form aggregates, resulting in increased flow resistance and high viscosity. At high shear rates, the shear thinning behavior of the system was related to the orientation of polysaccharide macromolecules along the streamline of the flow [26], as they gradually align themselves in the shear flow direction to reduce the resistance [27]. The polysaccharide aggregates formed at high concentrations may partially break at a high shear rate and strengthen the shear thinning behavior to a certain extent.

According to the flow curve, the flow properties of TSP solutions could be further described with the Williamson model [28] as follows:

(1) $$\eta =\frac{\eta_{0}}{1+{\left(\lambda_{w}\dot{\gamma }\right)}^{n}}$$

where *η* represents the apparent viscosity (Pa·s); *η*_0_ is the zero shear viscosity (Pa·s); $\dot{\gamma }$ the is shear rate (s^−1^); *λ_w_* represents the relaxation time (s); n is the shear-thinning index, where n < 1 indicates a shear-thinning behavior of polymers [29].

The fitted parameters of the Williamson model for TSP solutions are shown in Table 1. The model predictions could explain well the variations in the viscosity data (correlation coefficient R^2^ = 0.9164–0.9999). The solution behavior transformed from Newtonian to shear thinning at a critical shear rate (${\dot{\gamma }}_{w}$), which is the reciprocal of relaxation time (${\dot{\gamma }}_{w}$ = 1/*λ_w_*). The ${\dot{\gamma }}_{w}$ values of TSP solutions at the concentrations of 1.5%, 2%, and 4% were derived as 323, 132, and 21 s^−1^, respectively. The decrease of ${\dot{\gamma }}_{w}$ value with increasing concentration could be attributed to a dynamically entangled network structure in the solutions, limiting the movement of polymer chains under concentrated conditions [29]. The shear-thinning index (n) increased with increasing concentration from 0.659 to 0.772, which means the enhancement of shear thinning behavior and typical pseudoplastic fluid property of the solution system. The zero-shear viscosity *η*_0_ increased with the increase of concentration, indicating the establishment of a greater number of links between the biopolymer molecules, which depended on the molar mass and inter-chain interactions [30].

These results were consistent with most of the studies on rheological behaviors of polysaccharides such as xanthan gum [31,32] and -carrageenan [33]. These results indicated that TSP has potential applications in the processing of liquid products, since pseudoplastic fluids are favored for chewing, swallowing, as well as liquid filling. In addition, the time dependency is connected to the thixotropy concept at a constant shear rate. The measured shear stress or viscosity of a thixotropic material will decrease with time and finally stabilize to a constant value, which could be characterized by considering a ramp-up and ramp-down of shear imposed in a rotational rheometer [34]. Since the upward and downward curves overlapped quite well (Figure 3), it is speculated that the rate of structure breakdown is similar to that of its reconstruction [35]. Therefore, the time dependency could be neglected and ramps are suitable to describe rheological behavior of TSP solution.

### 2.4. Viscoelastic Behaviors of TSP

**Figure 4:**
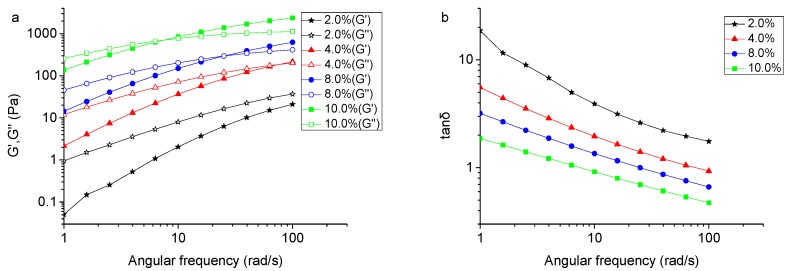
(**a**) Angular frequency dependencies of shear modulus G′(storage) and G″(loss) of TSP at different concentrations at 25 °C; (**b**) Angular frequency dependency of tan δ of TSP at different concentrations at 25 °C.

Polysaccharide solutions and gels are usually characterized by the storage modulus G′ and the loss modulus G″ to evaluate their viscoelastic properties. In general, if G′ < G″, the solution has prevalently viscous properties, which are observed to be more ‘‘liquid-like”. When G′ > G″, the system will exhibit prevalent elastic properties [11,36,37,38]. Figure 4 presents the viscoelastic behaviors of TSP solutions at different concentrations at a strain of 2%, which is chosen by the linear viscoelastic region checked in Figure S2. As shown in Figure 4a, the loss modulus G″ and storage modulus G′ of TSP solution increased with increasing frequency. For 2% (*w*/*v*) TSP solution, the G′ was lower than the G″ in the tested frequency range, indicating a fluidic behavior. When the concentration increasing to 4%, 8%, and 10% (*w*/*v*), the G′ and G″ increased and crossed at a frequency of 80, 25 and 7 rad/s, respectively. This indicates that the TSP solution transformed from viscous behavior to a more elastic behavior with increasing concentration [39]. The TSP solutions of all concentrations presented the entanglement behavior of typical uncharged random coil polysaccharides [40].

The mechanical loss of the TSP solution during oscillation measurements can be reflected by loss tangent (tan δ = G″/G′), which is a measure of the energy lost compared to energy stored in a cyclic deformation [41]. When tan δ = 1, the same degree of viscous and elastic components can be expected for the systems with typically weak gel properties [37]. As shown in Figure 4b, the system revealed liquid-like properties indicated by an increased tan δ with decreasing TSP concentration. When the concentration increased up to 4%, tan δ reduced to less than 1 in high frequency region, suggesting that the TSP solution formed a weak gel.

### 2.5. Effect of pH on the Rheological Property of TSP

**Figure 5:**
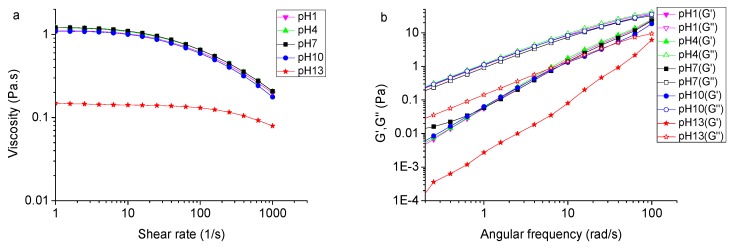
(**a**) Effect of pH on the apparent viscosity of TSP at the concentration of 2% (*w*/*v*) at 25 °C; (**b**) Effect of pH on dynamic modulus of TSP at the concentration of 2% (*w*/*v*) at 25 °C.

The apparent viscosities of TSP solutions at different pH values ranging from 1 to 13 are presented in Figure 5a. The results showed that the apparent viscosity of TSP was almost unchanged at pH 1–10, but decreased significantly at pH 13. It could be speculated that, in extreme alkaline solutions, the negative repulsion in the solution may destroy the intramolecular and intermolecular interactions of TSP, causing the apparent viscosity to decrease. It was perceivable that TSP solutions were stable in acidic and weakly alkaline environments. This is in contrast to Alpizar-Reyes’s [27] research, in which the apparent viscosity of TSP decreased with the decreasing pH. The decreasing viscosity may be due to an increase in charge density [42], which promotes a contraction of the TSP molecules. As reported, the apparent viscosity of xanthan is greatly influenced under acidic conditions, and was much lower at pH 3 than that at pH 5. It may be due to the increase in stability of the helical conformation of xanthan, decreasing the hydrodynamic volume size [43]. The viscosity of xanthan solutions exhibits a slight change in an alkaline environment [44]. The pH is an important gelation factor for pectin, which is an anionic polysaccharide. Lowering the pH value protonates carboxylic groups, reducing electrostatic repulsions along and between pectin chains [45]. Figure 5b shows the dynamic rheological properties of TSP (2% *w*/*v*) in different pH solutions. It shows that the G″ was higher than G′ over the entire frequency range and at different pH values. No intersection between the G′ and G″ curves implies that the pH change destroyed the weak gel structure of TSP, giving a viscous system.

### 2.6. Effect of Temperature on the Rheological Properties of TSP

**Figure 6:**
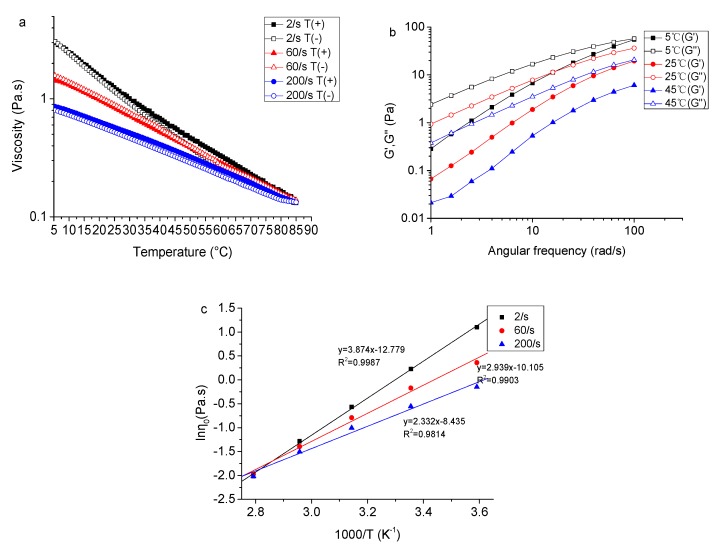
(**a**) Effects of temperature on the apparent viscosity of TSP (2%, *w*/*v*) at different shear rates; (**b**) Effects of temperature on dynamic modulus of TSP (2%, *w*/*v*) at 2 s^−1^; (**c**) Arrhenius plots for apparent viscosities of TSP solution (2%, *w*/*v*) at different shear rates.

The apparent viscosity changes of 2% (*w*/*v*) TSP solution with temperature are shown in Figure 6a. It is evident that the apparent viscosity of the TSP solution decreased gently with increasing temperature (5–85 °C) at three different shear rates. The apparent viscosity of TSP at a shear rate of 2 s^−1^ decreased from 3.01 to 0.13 Pa.s as the temperature increased from 5 to 85 °C. It could be speculated that TSP adopts a partial self-associated conformation in water due to hydrogen bonding and polymer entanglements at low temperatures. As the temperature increased, a transition progressively took place, from a partially ordered random broken helix conformation to a disordered random coil conformation [46]. As a result, the liquid-like properties of TSP solutions increased with increasing temperature, which was consistent with a previous report [27]. This is similar to the result reported for xanthan gum, of which the viscosity decreases rapidly from 785 to 0.746 Pa.s as the temperature increases from 20 to 80 °C [47]. Furthermore, during the heating-cooling process, the two apparent viscosity curves coincided well, indicating the good thermal resilience of TSP. This may be explained by the electrostatic interactions among polysaccharide chains and the stability of its hydrogen bonds [48].

The effects of temperature on storage modulus G′ and loss modulus G″ of TSP are shown in Figure 6b, where G′ and G″ declined with the increase in temperature (from 5 to 45 °C). At 5 °C, the curves of G′ and G″ crossed over at a frequency of 100 rad/s, while G″ values were higher than G′ in the tested frequency range at 25 °C and 45 °C. These results indicate that a TSP solution of 2% (*w*/*v*) tended to present a gel-like behavior at low temperature (5 °C), and a liquid-like one at high temperature (45 °C).

The apparent activation energy (Ea) for viscous flow was applied to investigate the temperature dependence of TSP solutions. Ea can describe the influence of absolute temperature on several chemical and physical processes according to the Arrhenius’ law (Equation (2)) [49]. The equation is as follows:

(2) $$\eta =\eta_{0}\exp\frac{\mathrm{Ea}}{\mathrm{RT}}$$

where *η* is the apparent viscosity (Pa.s); *η*_0_ is a pre-factor (Pa.s); R is the ideal gas constant (8.314 J·mol^−1^·K^−1^); T is absolute temperature (K); and Ea is the activation energy (kJ/mol) for viscous flow [50].

In this model, by plotting of lnη against the inverse of temperature (1/T), a straight line could be obtained to calculate the apparent activation energy of 2.0% (*w*/*v*) TSP solution as shown in Figure 6c. Table 2 lists the activation energies for viscous flow of 2.0% (*w*/*v*) TSP solution, which were 32.208, 24.435, 19.388 kJ/mol at the shear rates of 2, 60, or 200 s^−1^, respectively. It is indicated that the temperature dependence of the apparent viscosity of TSP solutions was gradually lessened with the increase in shear rate.

### 2.7. Effect of Salts and Sucrose on the Rheological Property of TSP

**Figure 7:**
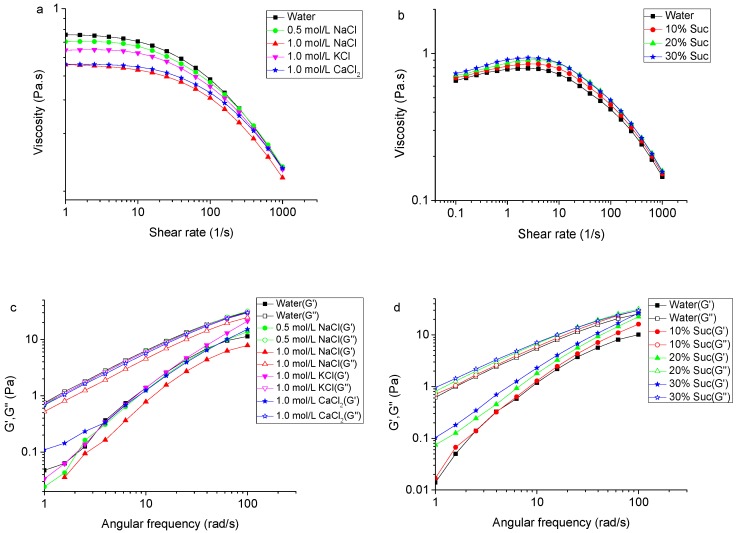
(**a**) Effect of Na^+^, K^+^, Ca^2+^ on the apparent viscosity of TSP (2%, *w*/*v*) at 25 °C; (**b**) Effect of sucrose on the apparent viscosity of TSP (2%, *w*/*v*) at 25 °C; (**c**) Effect of Na^+^, K^+^, Ca^2+^ on dynamic modulus of TSP (2%, *w*/*v*) at 25 °C; (**d**) Effect of sucrose on dynamic modulus of TSP (2%, *w*/*v*) at 25 °C.

The effects of Na^+^, K^+^, Ca^2+^ ions, and sucrose on the rheological properties of TSP are shown in Figure 7. The results reveal that the presence of these salt ions decreased the apparent viscosity to an extent depending on the ion type (Figure 7a). However, sucrose did so inversely and slightly (Figure 7b). TSP solutions maintained their shear thinning behavior in the presence of salt ions or sucrose. As shown in Figure 7c, different ions had little effects on the viscoelasticity of the 2% TSP solution. When TSP was dissolved in 1.0 mol/L NaCl solution, the G′ and G″ decreased to an extent greater than the other ionic solutions. Figure 7d shows the effect of sucrose concentrations on the viscoelastic moduli of 2% TSP solution. The G″ was higher than G′ over the entire frequency range in sucrose concentrations, implying that the solution was viscous (liquid-like) rather than elastic (gel-like). The decrease of apparent viscosity of TSP by the addition of Na^+^ might be due to the charge screening of electrostatic repulsions of the trisaccharide side-chain, which led to a more compact conformation and a reduction in the hydrodynamic size of the molecule [43]. The apparent viscosity of xanthan also decreased in the presence of Na^+^ [43]. However, K^+^ can enhance the apparent viscosities of xanthan gum and gellan solutions, since the association of charged helical structures was facilitated in different ways [38,51]. The effects of sucrose on polysaccharides may be due to the fact that sucrose reduced the free water of the system, enhancing the interactions between the polysaccharide chains, and therefore contributing to the viscosity of the solution system [43,52]. An apparent viscosity increase with increasing sucrose concentration is also observed for highly methoxylated (HM) pectin [53] and xanthan gum [52].

## 3. Materials and Methods

### 3.1. Materials

Tamarind seeds were collected from Yunnan, China. Monosaccharide standards (l-rhamnose, l-fucose, d-glucose, d-xylose, d-galactose, d-mannose, d-arabinose, d-glucuronic acid, and d-galacturonic acid) were purchased from Sigma-Aldrich Co. (St. Louis, MO, USA). Ethanol, phenol, citric acid, boric acid, acetonitrile, NaNO_3_, trifluoroacetic acid TFA, HCl, H_2_SO_4_, NaOH, KBr, NaCl, KCl, CaCl_2_ and sucrose were purchased from Yuanye Bio-Technology Co. (Shanghai, China). All reagents used were of analytical grade unless otherwise specified.

### 3.2. Extraction of TSP

Peeled tamarind seeds were firstly milled into 80-mesh flour, and then treated with 80% ethanol (1:10 *w*/*v*) at 25 °C for 6 h. The residue was collected by centrifugation (8000× *g*, 20 min) and then dried. A portion (10.0 g) of the treated sample was extracted with citric acid solution (solid-liquid ratio of 1:40, pH 3.5) with constant stirring at 80 °C for 20 min. After centrifugeation (8000× *g*, 20 min), the supernatant was collected and precipitated with ethanol at the ratio of 1:1 (*v*/*v*), and kept at 4 °C for two hours. The precipitate was then vacuum dried to a constant weight. The yield of TSP was determined by the following equation:

(3) $$TSP\;yield\;/\%=\frac{TSP\;weight}{raw\;materical\;weight}\;\times \;100$$

### 3.3. Chemical Components Analyses

Total carbohydrate content was determined by the phenol-sulfuric acid method [54] using glucose, xylose, and galactose in a molar ratio of 3:2:1 as the standard. Uronic acid content was measured by the m-hydroxybiphenyl method using d-galacturonic acid as the standard [55]. Total protein content was tested by the Kjeldahl method with a protein conversion factor of 6.25. Moisture and ash content were determined according to the AACC 44-15A and AACC 08-01 method [56], respectively.

### 3.4. Determination of Molecular Weight

Molecular weight (Mw) of TSP was determined by high performance size exclusion chromatography (HPSEC) employing Ultrahydrogel™ 500 and Ultrahydrogel™ 2000 columns (Φ 7.8 mm × 300 mm, Waters, Milford, MA, USA). The TSP sample (2 mg/mL) was prepared with 0.1 mol/L NaNO_3_ and filtered through a 0.45 μm microporous membrane. The mobile phase was 0.1 mol/L NaNO_3_. The elution was carried out at a flow rate of 0.6 mL/min and column temperature of 35 °C. Dextrans of known Mw (Mw 5,250; 13,050; 36,800; 64,650; 135,350; 300,600 and 2,000,000 Da) were used for calibration.

### 3.5. Determination of Monosaccharide Compositions

The monosaccharide composition of TSP was determined on a HPLC system coupled with a UV detector (Waters) via pre-column derivatization with 1-phenyl-3-methyl-5-pyrazolone (PMP) according to Strydom’s study [57] with several modifications. Reference monosaccharides, including rhamnose, arabinose, galactose, glucose, mannose, xylose, fructose, glucuronic acid and galacturonic acid were used to identification and quantification of samples. Samples were firstly hydrolyzed by 4 M trifluoroacetic acid (TFA) at 110 °C for 2 h. The hydrolysates were derivatized with PMP in an alkaline environment at 70 °C for 100 min. The derivatives were then neutralized with hydrochloric acid and dried under a stream of nitrogen. Chloroform and water were added to extract the PMP derivatives. This was repeated three times. The chloroform extracts were combined and filtered through a 0.45 μm membrane. A C18 column (Φ4.6 mm × 250 mm, XBbridge™) was used to separate the derivatives. The mobile phase was the mixture of acetonitrile and 0.1 mol/ L phosphate buffer (pH 6.7) with the volume ratio of 17:83 at a flow rate of 0.8 mL/min.

### 3.6. FT-IR Spectroscopy

The samples were analyzed using FT-IR (Vertex 70, Bruker, Karlsruhe, Germany) with a spectral range of 400–4000 cm^−1^ and resolution of 16 cm^−1^. The transmission of the samples was measured in 7 mm diameter KBr pellets.

### 3.7. Rheological Behavior of TSP

TSP samples were dissolved in distilled water at a series of concentrations of 0.5%, 1.0%, 1.5%, 2.0%, 4.0%, 8.0%, and 10.0% (*w*/*v*) (pH 6.87 ± 0.02). The effects of salt ions (0.5 M NaCl, 1.0 M NaCl, 1.0 M KCl, 1.0 M CaCl_2_) and sucrose (10%, 20%, 30% *w*/*v*) on the rheological parameters of TSP were conducted at a concentration of 2.0% (*w*/*v*) TSP. The samples at 2.0% (*w*/*v*) were also studied using different pH buffer solution (pH 1, 4, 7, 10, and 13). All the samples were prepared by stirring for 1 h at 80 °C and then resting for 12 h before analyses.

Rheological characterization was carried out on a Discovery HR-3 rheometer (TA Instruments) with a stainless flat plate geometry (diameter = 40 mm). The measuring gap was set as 1000 μm. The temperature was controlled by a circulating water device, which was coupled to the Peltier system (a temperature control system). During the temperature variation experiments, the system was covered with a thermal insulation cover to prevent water evaporation. Before all rheological measurements, samples were loaded onto the plate of the rheometer and allowed to reach equilibrium (±0.5 °C) for 5 min. Viscosity curves were obtained by applying an increasing shear rate (1–1000 s^−1^) and a decreasing shear rate (1000–1 s^−1^) at 25 °C. Viscoelastic behaviors of samples were evaluated with respect to angular frequency (0.1–100 rad/s) at a strain of 2% at 25 °C. For the thermostability study, temperature sweeps were performed by heating from 5 to 85 °C, and subsequent cooling from 85 to 5 °C at 5 °C/min and at a constant shear rate (2 s^−1^, 60 s^−1^, 200 s^−1^).

### 3.8. Statistical Analysis

The TRIOS software (Version 3.3.0.4055), which plugged with several fitting models (such as Carreau, Carreau-Yasuda, Williamson and Cross), was employed for the rheological data. ORIGIN 8.6 software was employed to process the rheological results. All experiments were done in triplicate. The differences between data were analyzed by Tukey’s tests and considered different at a significant level of *p* < 0.05.

## 4. Conclusions

This study investigated the chemical components and rheological properties of the polysaccharide from tamarind seed collected from Yunnan, China. The main monosaccharides of TSP with a Mw of 1735 kDa were glucose, xylose and galactose at a molar ratio of 2.9:1.8:1.0. TSP presented pseudoplastic behavior in water solution. The pH-resistance of TSP solution viscosity was excellent in the range of pH 1–10. The apparent viscosity of the TSP solution decreased with increasing temperature and ion concentration, but increased with sucrose concentration. Temperature, pH, salt ions, as well as the sucrose concentration were identified to slightly affect the rheological properties of TSP. With the aim of investigating the application of TSP in acidic beverages, high temperature processed foodstuffs, high-salt and high-sugar foods as thickener and stabilizer, further studies will focus on the interaction of TSP with food components.

## Figures and Tables

**Table 1:** Fitting parameters of Williamson model for TSP solutions at a series of concentrations.

Concentration (%, *w*/*v*)	*η*_0_ (Pa·s)	*λ_w_* (s)	n	R^2^
0.5	0.009	0.00003	0.659	0.9686
1.0	0.057	0.0007	0.699	0.9164
1.5	0.251	0.003	0.741	0.9999
2.0	0.953	0.008	0.753	0.9997
4.0	13.843	0.047	0.772	0.9999

**Table 2:** Fitting parameters of Arrhenius equation for 2% (*w*/*v*) TSP solutions.

$\dot{\gamma }$ (s^−1^)	ln*η_0_* (Pa.s)	Ea (kJ/mol)	R^2^
2	−12.778	32.208	0.9987
60	−10.106	24.435	0.9903
200	−8.435	19.388	0.9814

## Supplementary Materials

The following Supplementary Materials are available online: Figure S1: The elution curve of PMP derivatives of mixed monosaccharide standards hydrolysate by HPLC. Figure S2: Strain dependency of shear modulus G′(storage) and G″(loss) of TSP at different concentrations at 25 °C.
