# Supplementary material for: Composition and Rheological Properties of Polysaccharide Extracted from Tamarind (Tamarindus indica L.) Seed
Source: Molecules. 2019 Mar 28;24(7):1218. doi: 10.3390/molecules24071218 (PMC6480175; doi:10.3390/molecules24071218)
Supplement: Supplementary file 1 [file molecules-24-01218-s001.pdf]

Supplementary Materials

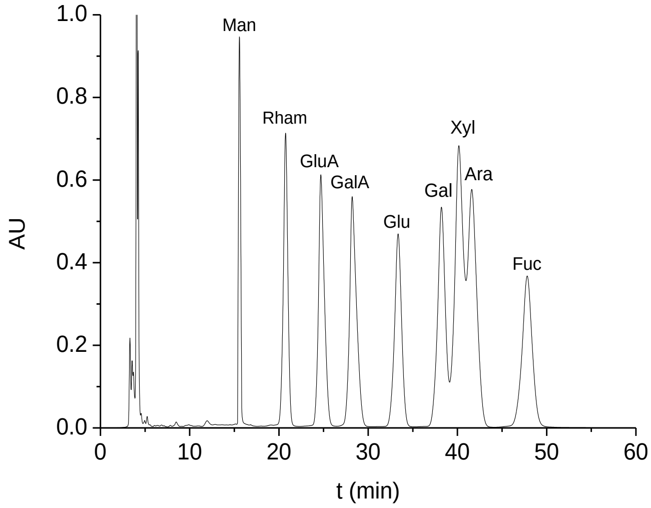

Figure S1. The elution curve of PMP derivatives of mixed monosaccharide standards hydrolysate by HPLC

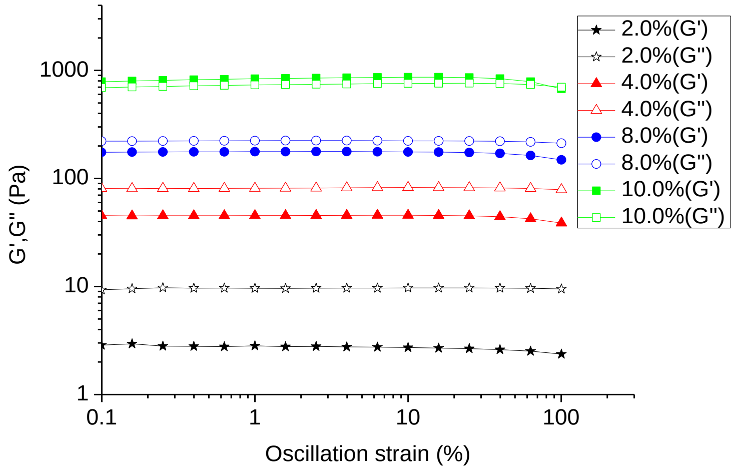

Figure S2. Strain dependency of shear modulus G'(storage) and G''(loss) of TSP at different concentrations at 25 °C
